# Patient Aesthetic Satisfaction with Timing of Nasal Fracture Manipulation

**DOI:** 10.1155/2014/238520

**Published:** 2014-01-02

**Authors:** Sunil Dutt Sharma, Ivor Kwame, John Almeyda

**Affiliations:** Department of Ear, Nose and Throat Surgery, West Middlesex University Hospital, Twickenham Road, Isleworth, Middlesex TW7 6AF, UK

## Abstract

*Introduction.* To determine patient cosmetic satisfaction following nasal fracture manipulation under general anaesthetic when offered at different time intervals after injury. *Materials and Methods.* Prospective chart review of adult patients with nasal fractures treated by closed reduction at a busy district general hospital in Greater London over a 10-month period. Patients were asked by a standardised telephone interview about satisfaction with nasal cosmesis pre- and postoperatively using a Likert scale. *Results.* Seventy-six of 106 patients presented for nasal manipulation at up to 9 weeks after injury and were successfully contacted (72%) postoperatively. Forty-nine patients (64%) reported that they still would have had the surgery in retrospect. Those done within 1-2 weeks after injury resulted in the highest mean satisfaction score (4.56 ± 0.25). There was a negative correlation between patient satisfaction and timing of surgery (*ρ* = −0.37, *P* = 0.001). Of the patients satisfied or very satisfied with their procedure, 96% had it done within 4 weeks. *Conclusion.* The majority of patients treated with closed reduction of nasal fractures under general anaesthetic are satisfied with the cosmetic outcome and would still have undergone surgery in retrospect. Increasing time of surgery after 2 weeks resulted in lower patient satisfaction.

## 1. Introduction

Nasal bone fractures are the most common type of facial bone fractures and indeed are one of the most common reasons for patients being referred to ENT surgeons. As the nose is the most prominent part of the face, it is the most susceptible to damage from facial trauma; Illum et al. noted that 39% of facial trauma involves the nose [1].

Nasal fractures are commonly caused by assaults, sports injuries, and road accidents. As they are often associated with multiple trauma, frequently nasal fractures are not promptly diagnosed and treated, leading to secondary nasal deformities and chronic obstruction, requiring more extensive procedures including septorhinoplasty [2].

Whilst there is published literature regarding the management of nasal fractures, there is no uniform policy or protocol for the management of this condition [3]. Standard practice in the United Kingdom is closed reduction within approximately 2 weeks of the injury [4]. However, increasing evidence shows persistent aesthetic concerns regarding cosmetic outcome and obstructive symptoms [5].

The aim of our study was to evaluate the cosmetic satisfaction of nasal fracture manipulation under general anaesthetic relative to the timing of surgery after injury. 

## 2. Material and Methods

A prospective evaluation was made of the medical records of all patients who, having been consented and counseled in the same manner, underwent surgical nasal facture manipulation under general anaesthetic via a standard technique (depression/elevation of any displaced bones and restoration of the midline alignment of the nasal pyramid) between April 2011 and March 2012 at the Ear, Nose and Throat Surgery Department of West Middlesex University Hospital in London. 

Patient demographics and timing of surgery after injury were recorded. A telephone interview was undertaken preoperatively and 1 month after surgery, which consisted of two sections: (1) patients' subjective opinion as to their cosmetic satisfaction (patients were asked “How would you rate your satisfaction with the appearance of your nose”) and (2) whether, in retrospect, patients would still have their operation. A Likert scale was used with a 5-point numerical scale to quantify the degree of satisfaction, with 5 being very satisfied and 1 being very dissatisfied. We also recorded surgeon's opinion of the success of the operation at one week after injury when the patient attended for splint removal. 

Statistical analysis of the difference between pre- and postoperative scores of nasal cosmesis and of the difference between responders and nonresponders was carried out using the Fisher's test for significance. Analysis of any correlation between timing of surgery after injury and cosmetic satisfaction was carried out using Spearman's rank correlation coefficient, *ρ* (GraphPad Software, USA) [6].

## 3. Results

106 patients underwent nasal fracture manipulation under general anaesthetic during the study period. Of these patients, 76 (72%) were successfully contacted for telephone survey. The majority of these patients were male (55 patients, 72%) and the mean age was 26.2 years (range, 16–56 years). Causes of nasal injury included assault (72%) and sports injuries (18%). Time to surgery varied because of delay in presentation of some patients to the ENT Department. 

When comparing responders (*n* = 76) and nonresponders (*n* = 30), there was no significant difference in male gender (55/76 versus 23/30, *P* = 0.81) or in age (54/76 under 30 years of age versus 24/30 under 30 years of age, *P* = 0.46).

Preoperatively, 4/76 patients (5%) were already satisfied or very satisfied with nasal cosmesis, whilst, one month postoperatively, 54/76 patients (71%) were satisfied or very satisfied with nasal cosmesis (*P* = 0.001), and thus there was a significant improvement in satisfaction postoperatively. Interestingly, 49/76 patients (64%) reported that they would still have had the surgery in retrospect (Table 1). 

Preoperatively, 38/76 patients (50%) were dissatisfied or very dissatisfied with nasal cosmesis, whilst, one month postoperatively, 11/76 patients (14%) were dissatisfied or very dissatisfied with nasal cosmesis (*P* = 0.001), and thus there was a significant reduction in dissatisfaction postoperatively. Of the patients who were dissatisfied or very dissatisfied with the surgery, 8/11 patients felt that they would like further revision surgery (representing 10% of the total population). Preoperatively, the mean score of patient satisfaction was 2.37 ± 0.80, whilst postoperatively the mean score of patient satisfaction was significantly higher (3.67 ± 0.86). 

**Figure 1:**
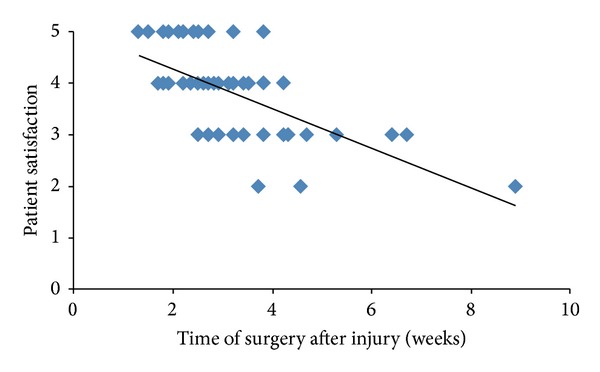
Relationship between timing of surgery after injury and patient satisfaction.

Surgical intervention between 1 and 2 weeks of injury resulted in a mean score of patient satisfaction of 4.56 ± 0.25, as opposed to a mean score of 4 ± 0.30 at 2-3 weeks and a mean score of 3.7 ± 0.37 at 3-4 weeks. At 8-9 weeks after injury the mean satisfaction score was 2 (Table 2). 52/54 patients (96%) who had surgery within 4 weeks of injury were very satisfied or satisfied with the cosmetic result (Figure 1).

The mean patient postoperative satisfaction score was higher in those patients whom the surgeon deemed had successful surgery at one week compared to the mean patient postoperative satisfaction score of those patients whom the surgeon deemed did not have successful surgery (3.82 ± 0.44 versus 2.98 ± 0.36). 

There was a negative correlation between patient satisfaction time of surgery and after nasal injury (*ρ* = −0.37, *P* = 0.001) (Figure 1). 

## 4. Discussion

In common with other studies regarding manipulation of nasal fractures under general anaesthetic, the majority of our patients were young males [7]. The most common cause for fractured noses in our region was assault, in contrast to other studies. This may be due to local socioeconomic factors in the region where our study took place in Greater London.

All patients within our study were operated by the same team of otolaryngologists, and therefore the technique of reduction was standardised to allow consistency amongst surgeons. The procedure involved the depression/elevation of any displaced bones and restoration of the midline alignment of the nasal pyramid, as described previously [8].

Our results showed that only 14% of patients were dissatisfied or very dissatisfied with the outcome of nasal fracture manipulation, with only 10% wanting further surgery. Previous studies describe rates of dissatisfaction from 14% to 50% [9]. In common with other studies, not all patients who were unhappy with the surgical outcome wished to have further surgery, probably due to the fact that they were not keen on further general anaesthetic and fear of complications of surgery [10].

Our centre is unique in that, in contrast to other centres, we operate a policy of offering closed manipulation of fractured noses to concerned patients even after an extensive interval after injury, provided the patient is fully willing to accept the potential risks and complications. Our results showed that an increased period of time between injury and surgical intervention demonstrated decreased satisfaction with cosmetic outcome. To our knowledge, there is no literature currently that has demonstrated this. We would therefore advocate early surgical intervention for those patients with a fractured nose, preferably within two weeks of injury. However, our results suggest that, even after 4 weeks, 95% of patients were satisfied or very satisfied with the cosmetic result, suggesting potential benefits of performing surgery up to 4 weeks after injury. 

The expression of satisfaction may be clouded by the desire not to undergo any further surgery [7]. One reason why only 64% of patients would have had surgery in retrospect may be related to fear of general anaesthetic, as opposed to perceived satisfaction with the operation, as has been previously described in the literature [9].

One of the limitations of this study was the fact that the Likert scale is subject to central tendency and social desirability bias; that is, patients may be more likely to give responses that they deem the assessor will want to hear. Unfortunately, it is very difficult to eliminate this sort of bias. However, in order to reduce this, we subjected our questionnaire to face validity using an “expert” group of consultants within the department to assess the robustness of the questionnaire. Although, to the authors' knowledge, there is no validated questionnaire to assess cosmetic satisfaction after closed fracture manipulation, a 5-point Likert scale similar to that used in this study has been successfully applied previously [7].

One may argue that one month after surgery may be too early for the patient to decide on satisfaction of the operation, thus limiting our study. Future work could perhaps look at satisfaction over a longer period after surgery and record clinicians' opinion of cosmetic result using the same Likert scale at one month. It may also be worth assessing patient satisfaction with functional outcome using validated questionnaires.

## 5. Conclusion

The majority of patients treated with closed reduction of nasal fractures under general anaesthetic are more satisfied with cosmetic outcome after surgery and would still have the surgery in retrospect. An increased length of time between injury and surgery results in decreased patient satisfaction, and thus we advocate early surgical intervention within two weeks of injury.

## 6. Summary

(i) ENT departments in the United Kingdom manage nasal fractures by offering closed reduction at a variety of different time intervals after injury.
 (ii) Our aim was to determine patient cosmetic satisfaction following nasal fracture manipulation under general anaesthetic when offered at different time intervals after injury.
 (iii) We assessed satisfaction with nasal cosmesis before and after surgery in patients who had surgical intervention at varying times after injury.
 (iv) Those patients who had surgery within 1-2 weeks after injury had the highest mean satisfaction score (4.56 ± 0.25), and there was a negative correlation between patient satisfaction time of surgery after nasal injury.
(v)  The majority of patients treated with closed reduction of nasal fractures under general anaesthetic are satisfied with the cosmetic outcome and would still have undergone surgery in retrospect, irrespective of the duration of time after injury, but increasing time of surgery after 2 weeks resulted in lower patient satisfaction.

## Figures and Tables

**Table 1:** Patient satisfaction with cosmesis before and after surgery.

Patient satisfaction	Frequency in preoperative group, *n*	Frequency in postoperative group, *n*
Very satisfied	0	12
Satisfied	4	42
Undecided	34	11
Dissatisfied	24	7
Very dissatisfied	14	4

**Table 2:** Relationship between timing of surgery after injury and mean patient satisfaction score (Likert scale).

Time of surgery after injury	Mean patient satisfaction score ± SE (Likert scale)
0-1 weeks	NA
1-2 weeks	4.56 ± 0.25
2-3 weeks	4 ± 0.30
3-4 weeks	3.68 ± 0.37
4-5 weeks	3.17 ± 0.42
5-6 weeks	3 ± 0
6-7 weeks	3 ± 0
7-8 weeks	NA
8-9 weeks	2 ± 0

NA: not applicable.

## References

[1] Illum P, Kristensen S, Jorgensen K, Pedersen CB (1983). Role of fixation in the treatment of nasal fractures. *Clinical Otolaryngology and Allied Sciences*.

[2] Mondin V, Rinaldo A, Ferlito A (2005). Management of nasal bone fractures. *American Journal of Otolaryngology*.

[3] Kapoor PK, Richards S, Dhanasekar G, Kumar BN (2002). Management of nasal injuries: a postal questionnaire survey of UK ENT consultants. *Journal of Laryngology and Otology*.

[4] Ridder GJ, Boedeker CC, Fradis M, Schipper J (2002). Technique and timing for closed reduction of isolated nasal fractures: a retrospective study. *Ear, Nose and Throat Journal*.

[5] Murray JAM, Maran AGD (1980). The treatment of nasal injuries by manipulation. *Journal of Laryngology and Otology*.

[6] http://www.graphpad.com/quickcalcs/contingency1.cfm.

[7] Hung T, Chang W, Vlantis AC, Tong MCF, van Hasselt CA (2007). Patient satisfaction after closed reduction of nasal fractures. *Archives of Facial Plastic Surgery*.

[8] Ondik MP, Lipinski BS, Dezfoli S, Fedok FG (2009). The treatment of nasal fractures: a changing paradigm. *Archives of Facial Plastic Surgery*.

[9] Crowther JA, O’Donoghue GM (1987). The broken nose: does familiarity breed neglect?. *Annals of the Royal College of Surgeons of England*.

[10] Fernandes SV (2004). Nasal Fractures: the training of the shrewd. *Laryngoscope*.

